# Venous-arterial extracorporeal membrane oxygenation for psittacosis pneumonia complicated with cardiogenic shock: case report and literature review

**DOI:** 10.1186/s12872-023-03669-y

**Published:** 2024-01-02

**Authors:** Yanting Zhang, Hongtao Hu, Ying Xu, Yi Chen, Biao Liu, Jun Chen, Wenfang Nie, Si Zhong, Jing Ma, Chang Liu

**Affiliations:** 1 Department of Critical Care Medicine, Zhongnan Hospital of Wuhan University, 430071 Wuhan, China; 2 Clinical Research Center of Hubei Critical Care Medicine, 430071 Wuhan, China

**Keywords:** VA-ECMO, Chlamydia psittacosis, Dilated cardiomyopathy, Case report, Literature review

## Abstract

**Introduction:**

Dilated cardiomyopathy (DCM) is characterized by the enlargement of the left ventricle or biventricular, accompanied by myocardial systolic dysfunction. Chlamydia psittacosis (CP) is a zoonotic pathogen, which can cause severe pneumonia, respiratory failure, and acute organ dysfunction. The deterioration of DCM caused by CP infection is extremely rare, and few cases of successful management were reported.

**Case presentation:**

We reported a 67-year-old male patient with DCM and chronic heart failure. Who was admitted to ICU with severe pneumonia, acute hypoxemic respiratory failure, acute decompensated heart failure, arrhythmia, and cardiogenic shock. Mechanical ventilation (MV) and venous-arterial extracorporeal membrane oxygenation (VA-ECMO) were established for respiratory and circulatory support. Broncho alveolar lavage fluid(BALF)was collected for culture and metagenomics next-generation sequencing (mNGS) test. Repeated mNGS tests indicated the high possibility of CP pneumonia, thereafter, moxifloxacin and doxycycline were prescribed. After targeted antibiotics and organ support treatment, pneumonia, respiratory and circulatory failure were gradually resolved, patient was successfully weaned from MV and VA-ECMO. Finally, the patient was recovered and discharged alive.

**Conclusions:**

Severe respiratory and circulatory failure caused by CP infection in DCM patients is a rare life-threatening clinical condition. Early accurate diagnosis, targeted antibiotic therapy, coupled with extracorporeal life support posed positive impact on the patient’s disease course and outcome.

## Introduction

Chlamydia psittacosis (CP) is a zoonotic pathogen [1], that can cause a range of symptoms upon infection, include fever, headache, muscle soreness, dry cough and dyspnea, etc. In severe cases, CP infection leading to the development of multiple organ dysfunction syndrome (MODS) [2–6].

Venous-arterial extracorporeal membrane oxygenation (VA-ECMO) is an extracorporeal life support technique that can provide circulatory and respiratory support [7], which could ‘buy’ time for etiological treatment and disease recovery. At present, VA-ECMO is widely used in pulmonary embolism, cardiac arrest, cardiogenic shock (CS), and bridging before heart transplantation [8, 9]. Here we present a case with dilated cardiomyopathy (DCM) and chronic heart failure, which exacerbated after CP infection, resulted in severe respiratory and circulatory failure, the diagnosis and treatment of this rare life-threatening clinical condition were reported here.

## Case report

**Fig. 1:**
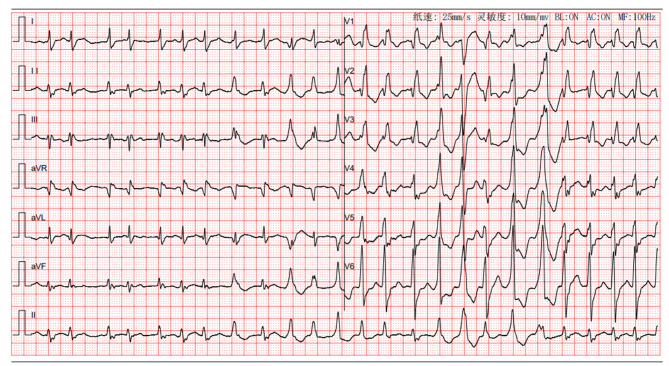
The patient’s electrocardiogram on admission showed a variety of arrhythmias, including atrial fibrillation, frequent premature ventricular contractions, complete right bundle branch block, and ST-T segment changes in certain leads

A 67-year-old male patient was transferred from the emergency department to ICU because of cough and wheezing for 5 days which worsened with dyspnea over the past day. The patient had a medical history of DCM, coronary atherosclerotic heart disease (CAHD), hypertension, and noninsulin-dependent diabetes mellitus (NIDDM), for over 10 years, but had not received standard and regular treatment. On admission, the patient was found to be unconscious, along with shortness of breath. Main abnormal physical examination findings included, blood pressure (BP), 68/56mmHg, heart rate (HR), 120–160 beats per minute, with an atrial fibrillation rhythm with frequent ventricular arrhythmias (as seen in Fig. 1), respiratory rate (RR), 30–40 breaths per minute, pulse oxygen saturation (S_P_O_2_), 88% (on the high-flow nasal cannula oxygen therapy [HFNO], with the FiO_2_ 100% and the flow rate 50 L/min), body temperature, 36.3℃. Extensive wheezing and crackles could be heard upon auscultation of both lungs.

**Fig. 2:**
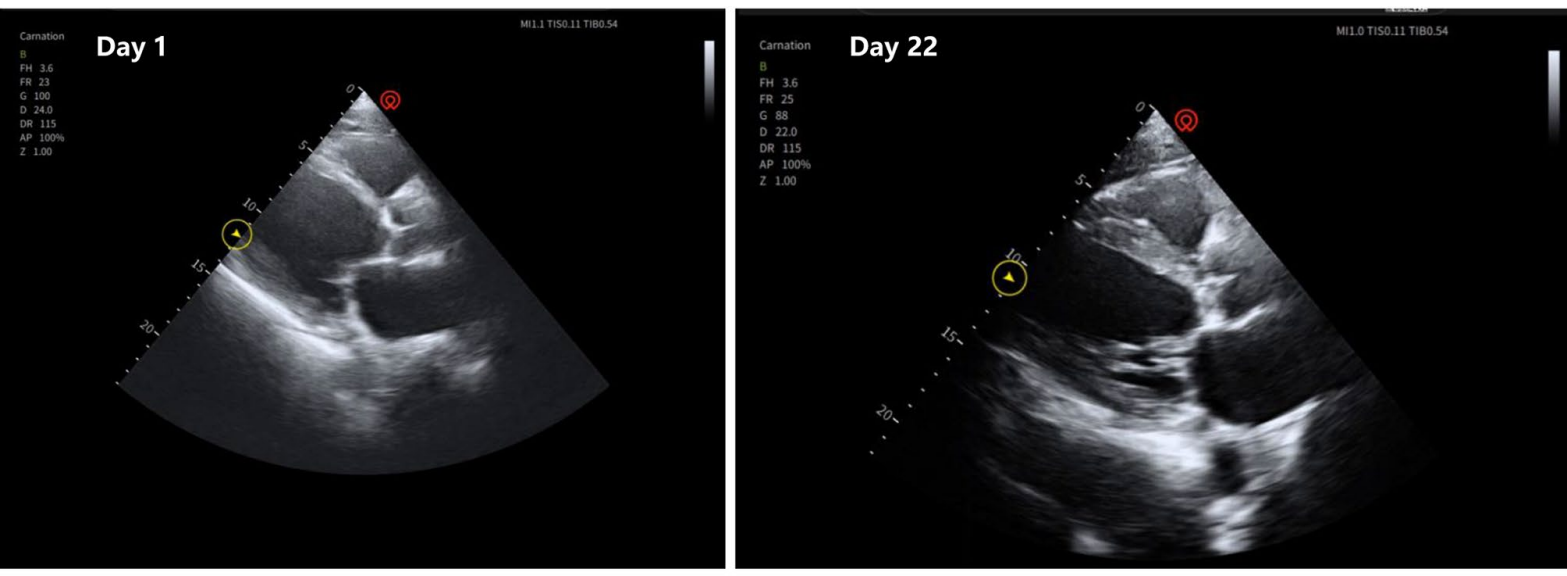
Comparison of echocardiography during the ICU stay. Day 1, the diameters of left atrial and ventricular were 5.7 and 7.0 cm, respectively, LVEF 19%. Day 22, the diameters of left atrial and ventricular were 5.3 and 6.3 cm, respectively, LVEF 32%

**Fig. 3:**
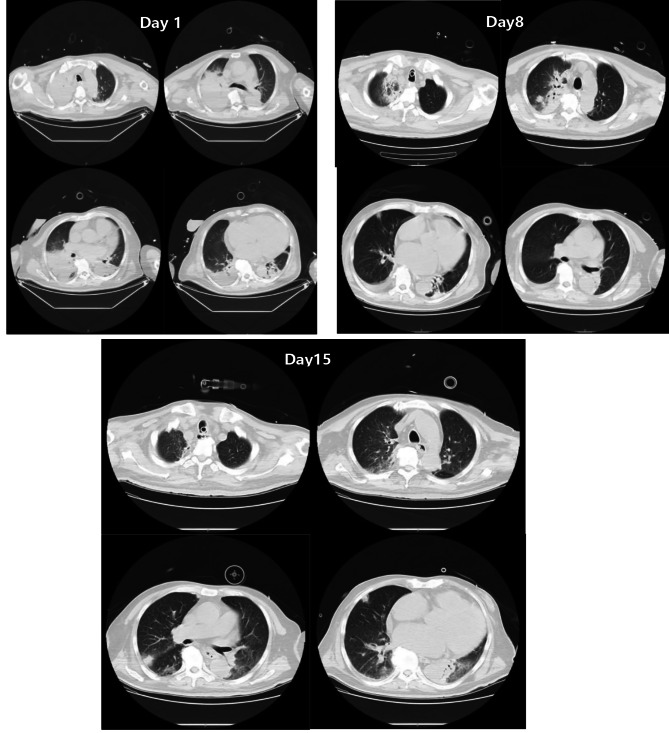
Comparison of the lung CT images of the patient during the ICU stay

**Fig. 4:**
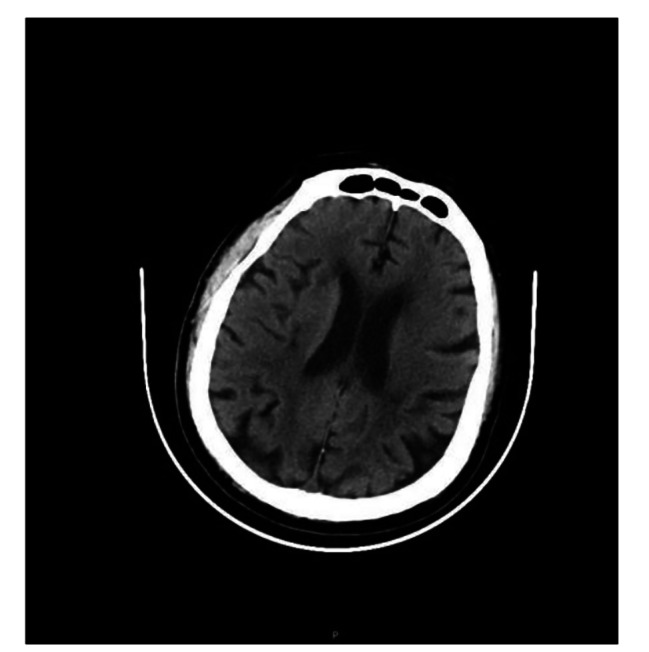
The patient’s brain CT scan on ICU admission

The arterial blood gas analysis (ABG) on admission indicated, pH 7.076, PaCO_2_ 65.1mmHg, PaO_2_ 45.5mmHg, lactate 6.8mmol/L, and HCO_3_- 13.3mmol/L. The laboratory tests revealed a white blood cell count (WBC) 8.5*10^9^/L, procalcitonin (PCT) level of 0.46ng/mL, interleukin-6 (IL-6) level of 267pg/ml, high-sensitivity cardiac troponin (HSTNI) level of 117.7pg/ml, and brain natriuretic peptide (BNP) level of 381.2pg/ml. Echocardiography showed the enlargement of the left atrium and left ventricular, the diameters were 5.7 and 7.0 cm, respectively, and the left ventricular ejection fraction (LVEF) decreased to 19%(Figure 2-Day1). Computed tomography (CT) scan demonstrated there were multiple infiltration and consolidation lesions in both lungs (Fig. 3-Day1), and no abnormalities in the head (Fig. 4). The APACHEII and SOFA scores on admission was 37 and 11, respectively. Upon admission the patient was diagnosed with severe community-acquired pneumonia, sepsis, acute hypoxemic respiratory failure, DCM, acute decompensated heart failure (ADHF), CS, atrial fibrillation with frequent ventricular arrhythmias, personal history of hypertension (classified as an extremely high-risk group), and NIDDM.

After ICU admission, the patient received HFNO and intermittent non-invasive mechanical ventilation (MV). Norepinephrine (NE) was administered to maintain blood pressure, furosemide, amiodarone, esmolol and levosimendan were administered to reduce cardiac load, control arrhythmia, and enhance myocardial contractility, respectively. After initial treatment, the patient’s clinical condition was still rapidly deteriorating, the dosage of vasopressors had been increased to NE 2.5ug/kg/min, terlipressin 0.03 IU/ kg/min, epinephrine 0.2ug/ kg/min, and the repeated ABG demonstrated, pH 7.066, PaCO_2_ 71.3mmHg, PaO_2_ 47.5mmHg, HCO_3_^−^ 14.1mmol/L, and lactate 7.0mmol/L.

As the conventional measures could not alleviate the clinical condition, treatments were upgraded to invasive MV, and VA-ECMO was quickly established through femoral artery-femoral vein catheterization to provide circulatory support. The rotational speed of ECMO was set at 7200 RPM with a flow rate of 3.7 L/min (Xenios Console, Medo Medizintechnik AG, Germany). Additionally, prone position was performed after the establishment of MV and VA-ECMO. Furthermore, fiber optic bronchoscopy examination was performed and BALF was obtained for culture and metagenomics next generation sequencing (mNGS) test.

With the assistance of MV and VA-ECMO, the patient’s oxygenation and circulation achieved significant improvement. However, high MV (PEEP 12cmH_2_O, FiO_2_ 60%) and VA-ECMO settings (flow rate 3.0-3.5 L/min), with a moderate dose of NE (0.5-1.0ug/kg/min) were persistently required. Additionally, the patient had a persistent fever, with body temperature fluctuating within 38.5–39℃, while empirical antibiotics was administered (piperacillin tazobactam). On the 3rd day of ICU stay, the mNGS test revealed the presence of CP gene fragments, but both of the BALF and blood cultures did not find any pathogens. To further confirm the etiology, another BALF sample was collected, BALF mNGS test, BALF and blood cultures were repeated. Meanwhile, antibiotics were adjusted to oral doxycycline and intravenous moxifloxacin.

After the adjustment, the patient’s body temperature, vasopressor dosage, ventilator and VA-ECMO settings gradually decreased. On the 5th day of ICU stay, the second mNGS test still reported the presence of CP gene fragments, yet the culture of blood and BALF were negative. Doxycycline and moxifloxacin continued to be used as targeted antibiotic therapy.

**Fig. 5:**
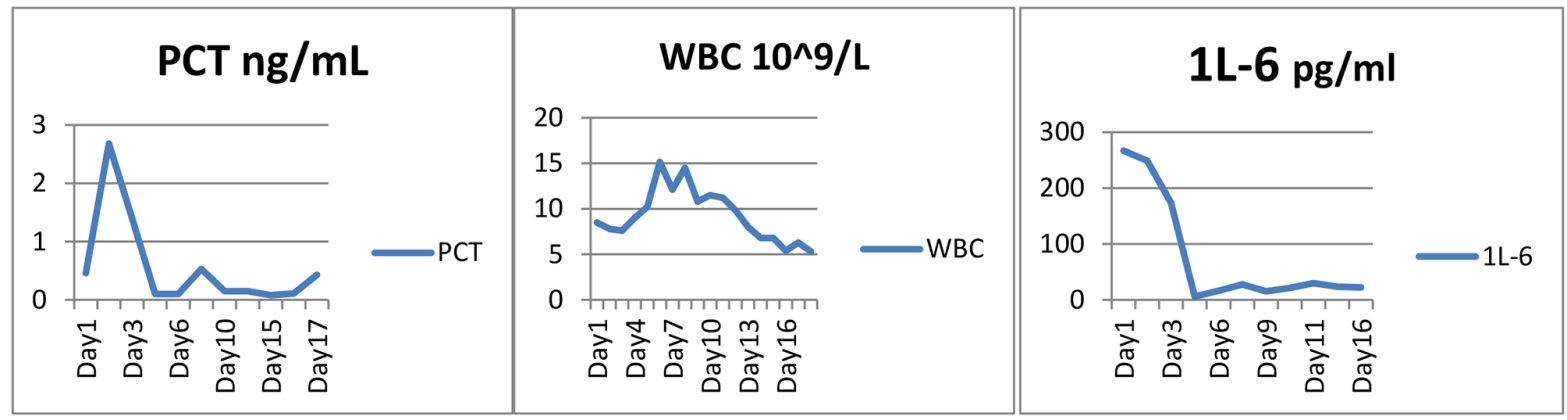
The test results of PCT, WBC, IL-6, HSTNI and BNP during the ICU stay

On the 7th day of ICU stay, the flow rate of VA-ECMO and the dose of NE were decreased to 1.7 L/min and 0.1ug/kg/min, respectively, thereafter, VA-ECMO was successfully withdrawn. On the 14th day of ICU stay, the patient’s consciousness was clear, and passed the spontaneous breathing test, MV and endotracheal tube were successfully discontinued and removed, respectively. Furthermore, the patient’s infection biomarkers gradually decreased (Fig. 5) and the lung CT scan demonstrated a gradual absorption of the infiltrative lesions (Fig. 3-Day8, Fig. 3-Day15). All antibiotics were stopped on the 16th day. Echocardiography demonstrated an improvement of cardiac function on the 22th day (Fig. 2-Day 22), the diameters of left atrial and ventricular were decreased to 5.3 and 6.3 cm, respectively, and the LVEF was increased to 32%.

On the 24th day of ICU stay, the patient’s vital signs were stable and the organ function were recovered to the baseline. The patient was transferred to the ward for further rehabilitation. Finally, the patient was recovered and discharged alive.

## Discussion

We reported a case of CP infection that resulted in the deterioration of DCM, severe ARDS, ADHF, CS and MODS. With the assistance of MV and VA-ECMO, patient was survived from the life-threating respiratory and circulatory failure, and the mNGS test facilitate the early etiological diagnosis. After targeted antibiotic therapy and organ support measures, patients were successfully weaned from extracorporeal life support measures and fully recovered.

CP is a pathogen known to cause zoonotic diseases. Although our patient did not acknowledge a direct contact with birds or poultry, we could not rule out the possibility that he had a direct or indirect contact with birds or individuals whohad CP colonization or infection, as his home besides a crowed traffic station and had a complex environmental exposure. In a study conducted by Zhang et al. on CP-related community-acquired pneumonia, it was found that CP could be transmitted from person to person, which was the first report on the human-to-human transmission of CP in China [10]. Although the epidemiological history was unclear, the mNGS test quickly and repeatedly identified CP gene fragments in the BALF samples, which strongly support the diagnosis of CP pneumonia. The mNGS does not rely on pathogen isolation and culture, and can directly perform non-targeted sequencing of sample DNA/RNA. It has the characteristics of rapid detection, high sensitivity, and wide coverage. Compared to conventional diagnostics, mNGS is less affected by the use of antibiotics, and sensitive to detecting rare, novel, and unexpected pathogens with no preconception [11]. The disadvantages of mNGS test including the high costs, potential false positive results, and the lack of the standardization of bioinformatics pipelines. In addition, we should also be careful when selecting the test samples for mNGS test. Studies have shown that compared to other samples, BALF sample yield the highest sensitivity and specificity, was especially appropriate for ICU patients [12]. Therefore, although the mNGS test demonstrated promising value in the diagnosis of rare infectious diseases, physicians must understand the potential benefits and drawbacks of mNGS test when applying it to clinical practice.

The main symptoms after CP infection in humans are respiratory symptoms. In this case, the patient initially experienced influenza-like symptoms such as chills and headache, which then rapidly progressed to pneumonia. Studies have shown that the average incubation period for CP infection is 5–14 days from asymptomatic to fulminant invasive disease, which can affect multiple systems and organs throughout the body [13]. In this case, the patient had the history of DCM and chronic heart failure, which were further deteriorated by CP infection, resulted in the life-threatening MODS.

Tetracycline antibiotics are the first choice for the treatment of CP infections. For mild to moderate CP infections, doxycycline or minocycline can be taken orally, while severe cases may require intravenous administration of doxycycline. Treatment with tetracycline usually takes effect within 24–48 h, such as a reduction in body temperature. It is important to continue the medication for at least 14 days, preferably up to 21 days, to prevent the risk of recurrence [14–16]. In this case, the patient responded well to the combination of oral doxycycline and intravenous moxifloxacin for more than 14 days. The targeted therapy showed positive clinical course, also supporting the diagnosis of CP infection.

In this case, an acute exacerbation of DCM led to refractory shock. Infection and shock are major risk factors for sudden cardiac death in patients with DCM [17]. Our patient had severe hemodynamic instability upon ICU admission, the top priority was to maintain organ perfusion and prevent multi-organ dysfunction at that time. However, after the initial treatment, including the respiratory support, reducing the cardiac preload, correction of acidosis and arrhythmia, along with the administration of high dose vasoactive drugs. The hemodynamics were still worsening, advanced respiratory and circulatory assist had to be considered. VA-ECMO is the most appropriate treatment for refractory shock, which drains venous blood through the central vein, oxygenates venous blood through the oxygenator and then pumps them into the aorta to maintain the perfusion and oxygenation of various organs, so that the heart and lung would get sufficient rest to recovery. VA-ECMO provides strong respiratory support in addition to circulatory support, and is especially suitable for patients with simultaneous circulatory and respiratory failure [18]. In this case, VA-ECMO was initiated quickly as the rapid deterioration of the patient’s hemodynamics and organ dysfunction. The early initiation of VA-ECMO provided great assistance for the hemodynamic stabilization and bought time for the etiological treatment.

## Limitation

There are some limitations in the management of this case. Firstly, the patient presented with hemodynamic instability and atrial fibrillation on admission, but we chose pharmacological cardioversion over electrical cardioversion as the first choice. Recent guidelines recommended that patients with atrial fibrillation and hemodynamic compromise should undergo immediate electrical cardioversion [19]. Our treatment may delay the correction of arrhythmia and the stabilization of cardiac output. Secondly, the patient received HFNO and non-invasive MV on ICU admission, although quickly switched to invasive MV. We had to acknowledge that, in our case who with severe acute hypoxic respiratory failure and ADHF, HFNO or non-invasive MV was not conducive to protecting the airway, maintaining the oxygenation and reduce the breathing load. However, there were certain advantages in the management of this case, such as the early identification of CP pneumonia via repeated mNGS tests, demonstrating the value of mNGS test in the diagnosis of rare infectious diseases. And the successful use of VA-ECMO in the management of acute exacerbation of DCM.

## Conclusion

Severe respiratory and circulatory failure in patients with DCM caused by CP infection is a rare life-threatening clinical condition. The mNGS test facilitated the etiological diagnosis, the combination of targeted antibiotic therapy, MV and VA-ECMO successfully rescue the patient’s live. Our case provided new diagnosis and treatment options for the management of similar patients in the future.

## Data Availability

The raw data supporting the conclusions of this article will be made available by the corresponding authors or the first author, without undue reservation.
